# Cluster analysis of *Plasmodium* RNA-seq time-course data identifies stage-specific co-regulated biological processes and regulatory elements

**DOI:** 10.12688/f1000research.9093.1

**Published:** 2016-08-08

**Authors:** Efejiro Ashano, Itunuoluwa Isewon, Jelili Oyelade, Ezekiel Adebiyi

**Affiliations:** 1 Covenant University Bioinformatics Research (CUBRe), Covenant University, Ota, Ogun State, 110001, Nigeria; 2 Department of Computer and Information Sciences, Covenant University, Ota, Ogun State, 110001, Nigeria; 3 Division of Applied Bioinformatics, German Cancer Research Center (DKFZ), Heidelberg,, 69120, Germany

**Keywords:** RNA-seq, Plasmodium, regulatory elements, expression profiles, malaria

## Abstract

In this study, we interpreted RNA-seq time-course data of three developmental stages of *Plasmodium *species by clustering genes based on similarities in their expression profile without prior knowledge of the gene function. Functional enrichment of clusters of upregulated genes at specific time-points reveals potential targetable biological processes with information on their timings. We identified common consensus sequences that these clusters shared as potential points of coordinated transcriptional control. Five cluster groups showed upregulated profile patterns of biological interest. This included two clusters from the Intraerythrocytic Developmental Cycle (cluster 4 = 16 genes, and cluster 9 = 32 genes), one from the sexual development stage (cluster 2 = 851 genes), and two from the gamete-fertilization stage in the mosquito host (cluster 4 = 153 genes, and cluster 9 = 258 genes). The IDC expressed the least numbers of genes with only 1448 genes showing any significant activity of the 5020 genes (~29%) in the experiment. Gene ontology (GO) enrichment analysis of these clusters revealed a total of 671 uncharacterized genes implicated in 14 biological processes and components associated with these stages, some of which are currently being investigated as drug targets in on-going research. Five putative transcription regulatory binding motifs shared by members of each cluster were also identified, one of which was also identified in a previous study by separate researchers. Our study shows stage-specific genes and biological processes that may be important in antimalarial drug research efforts. In addition, timed-coordinated control of separate processes may explain the paucity of factors in parasites.

## Introduction

In the past two decades, there has been an extraordinary commitment to the control and elimination of malaria which has resulted to a significant decrease in global malaria morbidity and mortality ( Rao, 2015). In the bid to finding new ideas to deal with the disease, we have witnessed a shift from orthodox independent studies in biochemistry and microbiology to using a more multidisciplinary and robust approach that embraces other fields such as computer science and mathematics. The so called “omics” science has been employed with some measure of success in malaria research ( Sarker *et al.*, 2013). Studies in the transcriptome of malaria causative parasites in both human and animal models have revealed the integral role of transcriptional regulation in *Plasmodium* biology. Taking advantage of the availability of the full genome sequence data of *P. falciparum*, the parasite's complex expression patterns through each developmental stage within its respective unique environment have been studied using microarray. More recently, whole transcriptome short-gun techniques (RNA-seq) have been shown to be a more accurate predictor of expression trends than micro arrays ( Wang *et al.*, 2009).

Finding cis-regulatory elements using *in-silico* methods assumes that genes that share similar expression trends ( *i.e.* that are “co-transcribed”) are likely to be controlled by a common regulatory element ( Young *et al.*, 2008). These potential regulatory elements with promoter functions can be found upstream of the expressed genes and will appear as conserved sequence motifs common to genes found in the same cluster, but scarce in the remainder of the genome ( Young *et al.*, 2008). Clustering methods, often used in the study of gene expression profiles, have also been applied to the analysis of time-course data for over a decade ( Eisen *et al.*, 1998; Lukashin & Fuchs, 2001). Clustering algorithms group gene expression profiles on the basis of a distance metric. Backed by the power of statistics, this approach has been used effectively as a tool for visualization of micro-array, and more recently, RNA-seq data in identifying groups of co-regulated genes ( Nueda *et al.*, 2014; Tarca *et al.*, 2006).

Presently, there is a very little information of the basal transcription machinery of Plasmodium species. Very few transcription factors have been identified and this is largely attributable to the AT-rich nature of the parasites genome which makes it difficult to identify regulatory elements within ( Callebaut *et al.*, 2005; Young *et al.*, 2008). There might be an underlying reason for the paucity of transcription factors however ( Subramaniam *et al.*, 2001).

Based on these motivations, we sought to analyze *Plasmodium* RNA-seq data to yield insight that could potentially be used in antimalarial drug discovery research. Our efforts primarily focused on identifying biological processes of therapeutic interest and implicated cis-regulatory elements involved in the coordinated regulation of these processes.

## Materials and methods

### Data

The data used in this study, made freely available by Otto and colleagues ( Otto *et al.*, 2014), comprised 5020 normalized gene fragments per kilobase of exon per million (FKPM) expression values of *Plasmodium berghei* ANKA. Two replicates of gene expression values were collected at six time points, these time points corresponding to the ring form (RI and RI-R), the trophozoite (Tr and Tr-R), schizont (Sch, Sch-R), gametocyte (G and G-R), 16-hour ookinete (O-16, O-16R) and the 24 hour ookinete (O-24h) morphological phases of the parasite life cycle. These morphological phases span three life-cycle stages commonly named the intra-erythrocytic development cycle, gametocytes/gamete stage, and the fertilization and ookinete development (the last stage which occurs within the vector host). For convenience, these stages are indicated as IDC, SEX and MOS. The IDC time-points included the ring form, trophozoite and schizont time-points; the SEX included the ring form, trophozoite and gametocyte time-points; and the MOS included the gametocyte, 16-hour ookinete and the 24-hour ookinete time-points respectively.

### Significant genes and clustering of profiles

Identification of statistically significant expressed (or repressed) genes (p < 0.05) and expression profile clustering for the IDC, SEX and MOS development stages of *P. berghei* was done in R (version 3.2.2) on a Windows 64-bit platform using the maSigPro package. maSigPro was initially designed for microarray time course experiments but has since been upgraded to handle next-generation sequencing (NGS) series data properly. It finds genes with significant temporal expression changes using a two-step regression strategy. For single time course experiment, the procedure first adjusts the global model by the least-squared technique to identify differentially expressed genes selecting significant genes with a false discovery rate (FDR) control procedure. The regression fit for each gene is defined by computing p-values associated to the F-Statistic of the model, which is used to select significant genes. P-values are corrected for multiple comparisons by applying the linear step-up (B-H) FDR procedure ( Benjamini & Hochberg, 1995). Finally, a stepwise regression is used to find statistically significant different profiles. Significantly expressed genes with similar expression patterns are then clustered using a hierarchical clustering approach applying the coefficients obtained in this second regression model. ( Nueda *et al.*, 2014). The cut-off value for the R-squared of the regression value used for this study was 0.7. The number of clusters was set as 9.

### Functional enrichment

Gene ontology (GO) analysis was performed with PlasmoDB ( http://plasmodb.org/plasmo/) which sources GO information from Interpro ( http://www.ebi.ac.uk/interpro/) and the Annotation Center. The top ranked enriched GO terms for biological processes were generally reported except in circumstances where no biological process was enriched with the gene set, in which case, GO terms for components were highlighted. A p-value cutoff was set to 0.05 (see supplementary file S01).

### Promoter motifs

The Suite for Computational identification Of Promoter Elements, “SCOPE” motif finder ( http://genie.dartmouth.edu/scope/) was used to predict candidate promoter motifs in this study. The SCOPE motif finder is designed to identify candidate regulatory DNA motifs from sets of genes that are coordinately regulated using three program algorithms ( Carlson *et al.*, 2007). A fixed upstream length of 1000 bp was maintained as default for *Plasmodium* species ( Harris *et al.*, 2011; Jurgelenaite *et al.*, 2009). Input genes used for the detection of motifs included *P. falciparum* (3D7) orthologues of the corresponding *P. berghei* genes extracted from PlasmoDB. Consensus sequences were generally reported if they had a coverage of greater than 90%, a greater than 2-fold ratio of motif count to gene number per cluster, and a significant (sig) value ( Carlson *et al.*, 2007) that was greater than 10. A sig value of 0 implies that one motif of that significance is expected by chance. Carlson *et al.* (2007) demonstrated that sig values performance on synthetic data significantly improved after the value of 10 and remained fairly unchanged below the sig value of 55.

## Results and discussion

### The IDC reveals that *Plasmodium* species expresses fewer genes when compared to other stages

MaSigPro identifies differentially expressed genes from FPKM normalized gene expression and selects genes that are significantly expressed applying false discovery rate control procedures. The data show that relatively fewer genes are up-regulated in the IDC ( Table 1). Only 1448 genes of the 5020 genes (~29%) captured in the time-course experiment showed any significant activity. Furthermore, between the three clusters that were up-regulated in this stage, only 155 genes out of the 1448 (~0.1%) have been functionally annotated or identified. Likewise, data from earlier studies that used different investigatory approaches seem to support this observation. Early experiments by McGarvey *et al.* (1984) in recombinant clones corresponding to genes expressed specifically during the late schizont-merozoite stage of *P. falciparum* development showed that the maturation of the parasite in this stage was associated with the selective activation of a relatively small set of genes. In another study, the level and nature of transcriptional activity in *P. falciparum* and its role in controlling gene expression during the IDC was investigated using nuclear run-on on whole-transcriptome analysis ( Sims *et al.*, 2009). In this experiment, it was observed that the total transcriptional activity involved in the IDC was seen to peak late in the stage at the advent of other morphological stages ( *i.e.* the SEX and MOS stage). A possible explanation to this was given in Bozdech *et al.* (2003) who suggested that *Plasmodium* species induce genes only when required and just once per cycle. Other possible reasons for this controlled regulation may be as an adaptive response by the parasite to evade the advanced immunological mechanisms of its human host and leaving the parasite with fewer targets for drugs.

**Table 1.** Number of significant (p < 0.05) genes found in the IDC, SEX and MOS developmental stages of *Plasmodium berghei* against the total number of sampled genes expressed in percentages (column 3).

Stage	Significant Genes	Percent
IDC	1448	28.84
SEX	2712	54.02
MOS	4383	87.31

### Clustering and GO analysis reveal unique biological processes up-regulated at particular times respective to each developmental stage

Genes can be clustered based on common function or sub-cellular complex membership, a process that has enjoyed some success over time. However, the functions of a large amount of the genes in *Plasmodium* are unknown, making this approach unsuited for a full analysis of the parasite’s genome especially for parasite-specific processes and sub-cellular structures. In addition, as seen in kinases and proteases involved in signal transduction, two genes of similar function may not share a common regulatory element, which further flaws this approach. On the other hand, steady state mRNA levels provide a more direct estimation of the transcriptional effects of cis-acting regulatory elements with genes of similar expression trends a better basis for identifying putative regulatory elements ( Young *et al.*, 2008; Zhou *et al.*, 2005). Young *et al.* (2008) used a semi-supervised clustering algorithm OPI that utilized information on *Plasmodium* gene function sourced from the GO database to guide clustering of genes based on microarray derived life cycle mRNA expression patterns. In this study however, we opted for an unsupervised hierarchical clustering algorithm that altogether eliminated the bias brought into the clustering process with the prior knowledge from gene ontology ( Herrero *et al.*, 2001). This resulted in grouping highly co-expressed genes in clusters based simply on similarities in calculated expression patterns independent of gene function or sub-cellular complex membership. Results of identified associated GO biological processes and components enriched in clusters of their respective developmental stages with reference to studies of these processes in malaria control are shown in Table 2. The importance of these processes in parasite development and the relevance in malaria control are discussed in more detail in the following paragraphs. It is worth noting that in a previous microarray study on the transcriptome of the intraerythrocytic development cycle of *Plasmodium falciparum*, it was observed that induction of genes occurred selectively at specific times and only when required ( Bozdech *et al.*, 2003). Our results not only further support this report, but also suggest that the same applies for other stages of the *Plasmodium* development cycle.

**Table 2.** Cluster identified in the IDC, SEX and MOS developmental stages of *Plasmodium berghei* showing respective associated GO terms and their background percentages. References of relevant processes targeted by on-going drug related research are shown in column 4.

Cluster	Associated GO terms	Background percent	REFERENCES
**IDC**			
Cluster 4 (16 genes)	Apical part of cell (GO:0045177)	5*	
Cluster 9 (32 genes)	Lipoate biosynthetic process (GO:0009107)	50	Storm & Muller (2012)
**SEX**			
Cluster 2 (851 genes)	Cell gliding (GO:0071976)	75	
	Vacoular transport (GO:0007034)	75	
	Regulation of actin filament length (GO:0030832)	57.1	
	Entry of host cell (GO:0030260)	52.4	
	Protein palmitoylation (GO:0018345)	50	
**MOS**			
Clusters 4 (153 genes)	Peptidyl-arginine N-methylation (GO:0035246)	100	Dillon *et al.* (2012)
	Regulation of cell proliferation (GO:0042127)	100	
	Protein processing involved in protein targeting to mitochondrion (GO:0006627)	100	
	Protein farnesylation (GO:0018343)	100	Eastman *et al.* (2006)
	U6 snRNA 3'-end processing (GO:0034477)	100	
Cluster 9 (258 genes)	Peptidyl-diphthamide biosynthetic process from peptidyl-histidine (GO:0017183)	66.7	
	Vesicle docking involved in exocytosis (GO:0006904)	50	

### Uncharacterized genes may provide alternative sources for drugs targets of the IDC

**Figure 1.**
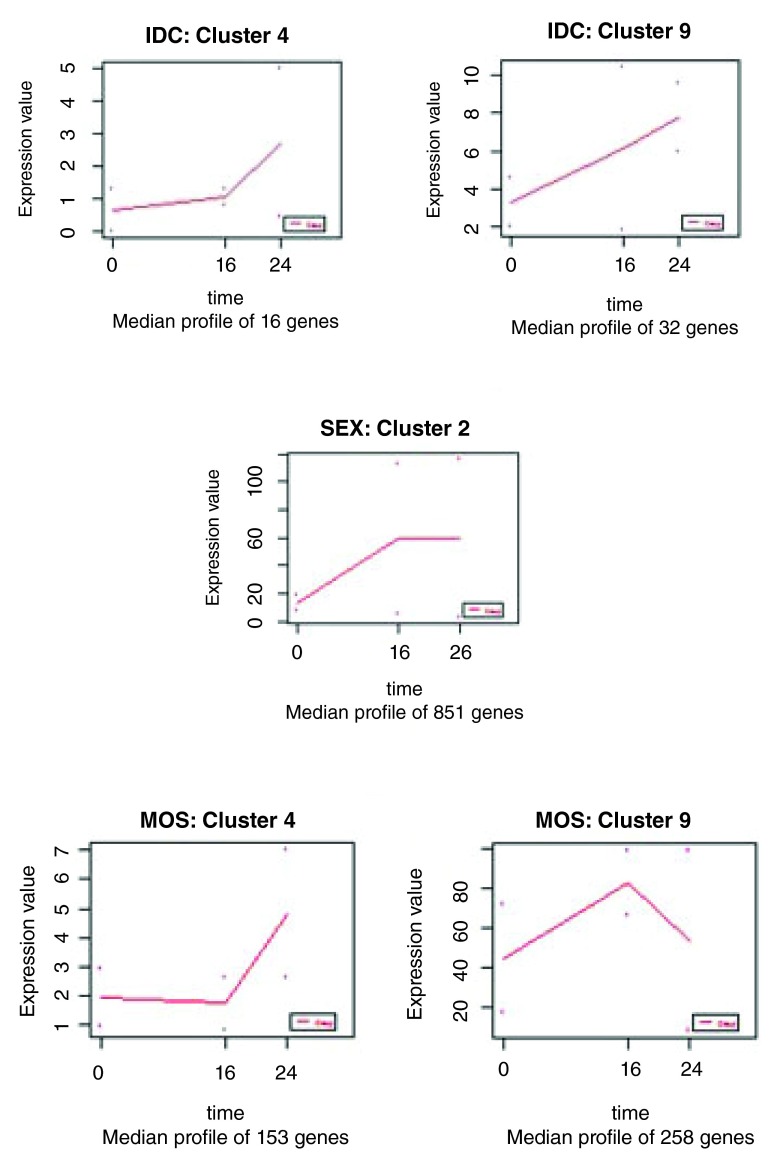
Selected median expression profiles of the clusters of the IDC (cluster 4 and cluster 9), SEX (cluster 2), and MOS (cluster 4 and 9) showing the number of genes of each gene-set.

It takes about 16 hours for a merozoite (via the ring form) to be transformed to a matured trophozoite just before it divides its nucleus. It takes another 6 – 8 hours for the schizogony to be complete unleashing a fresh set of meroziotes for reinvasion. Of the 5020 genes analyzed, a total of six clusters where identified to show up-regulated trends of fold changes greater than 2 from their median expression profiles in the three life stages of the parasite. For the IDC, two clusters showed significant up-regulated expression. These included cluster 4 (16 genes) which was up-regulated after the 16-hour time point; and cluster 9 (32 genes) which was up-regulated from the 0-hour time point up until the 24-hour time point ( Figure 1). GO analysis of these clusters showed the former to be associated with genes involved in the “apical part of the cell” (GO:0045177), while the latter being associated with the “lipoate biosynthetic process” (GO:0009107). Only 5% of the genes compared to the background of genes found in the apical part of the cells were identified in cluster 4 so it is unlikely that the apical part of the parasite plays a significant role in in this development stage. Some of the genes identified within cluster 4 may however be important therapeutic targets. These include the TREP (TRAP-like protein) (PBANKA_1306500), SIAP1 (PBANKA_1006200) and PLP4 (PBANKA_0711400) proteins among other highly conserved proteins which have not at the time of writing this report been characterized (see supplementary file S02). SIAP1 and TRAP have been implicated in the invasion of sporozoites by facilitating binding to host cells. Knock-out experimentation of TRAP in *P. berghei* led to non-motile sporozoites and by such, has been thought to be an important therapeutic target ( Ejigiri *et al.*, 2012; Greenwood *et al.*, 2008; Sultan *et al.*, 1997). Other studies also confirm the expression of this protein during the IDC ( Baum *et al.*, 2008). A list of highly conserved coding genes with unknown function that can be studied for function and as potential drug targets in this and other clusters can be found in the supplementary file S02. Our results also show that Lipoate-protein ligase (LipB), one of the two genes involved in Lipoic acid synthesis is steadily increased throughout the IDC period. Lipoic acid is an integral cofactor of α-keto acid dehydrogenase complexes and the glycine cleavage system, the metabolite playing a dual role in both intermediate metabolism and as a redox sensor/antioxidant. Biosynthesis of lipoate occurs exclusively in the apicoplast involving LipB and lipoic acid synthase. There is evidence however that disruption of the LipB gene does not negatively affect the growth of the parasite suggesting the protein’s redundancy. Lipoate scavenging is another route for lipoate acquisition which is critical for *Plasmodium* survival especially in the liver stages of development and may be a better target than the actual lipoate synthesis by the parasite for drugs ( Allary *et al.*, 2007; Günther *et al.*, 2009; Storm & Muller, 2012).

### Sustained up-regulated biological processes linked to invasion may present key drug targets to prevent transmission

In each IDC a few parasites loop out of asexual multiplication and differentiate into sexual cells, otherwise known as gametocytes. These haploid macro-gametocytes (females) and micro-gametocytes (males) are the precursor cells of the female and male gametes. Some evidence shows that the parasite commits to forming gametocytes 12 – 16 hours after invasion. The factors that cause trophozoites to differentiate into gametocytes in preference to schizonts are not known. It is known however, unlike in *P. falciparum*, they do not have periods of “pure” differentiation where “all” trophozoites differentiate into gametocytes. It should be of note that gametocyte formation is necessary for the transmission of *P. berghei* since this is the only form by which the mosquito can take up the parasite during a blood meal ( Landau & Gautret, 1998). In the SEX, two clusters showed regulatory patterns of interest. The median expression profile of cluster 2 (851 genes) was increased between the 0 hour and the 16 hour time points. This increase is seen to level off for the remaining part of the life-cycle stage. Cluster 5 (27 genes) also demonstrated an up-regulated expression profile, but only after the 16 hour time point ( Figure 1). GO analysis of cluster 2 in SEX showed that the biological process associated with this cluster includes "cell gliding" (GO:0071976), "vacuolar transport" (GO:0007034), "Cell motility" GO:0048870, "regulation of actin filament length" (GO:0030832), "entry into host cell" (GO:0030260), "protein palmitoylation" (GO:0018345). There was no GO term that was significantly (p > 0.05) associated with cluster 5. The significant processes however may be of some importance in this life-cycle stage. Previous investigations have demonstrated that invasion is dependent on the gliding and motility of the parasite which is dependent on the actin and myosin present in the parasite's pellicle ( Boysen & Matuschewski, 2013; Dobrowolski *et al.*, 1997; Dobrowolski & Sibley, 1996; Heintzelman & Schwartzman, 1997). Invasion of the parasite also involves vacuolar transportation by rhoptries. Rhoptries are club-shaped structures containing a long duct through which the luminal contents are extruded at the time of host cell invasion. Thought to be similar in function to multi-vesicular bodies (MVBs), they may function in sorting specific proteins and lipids. Unlike MVBs however, their membranes are characterized by an unusually high cholesterol – phospholipid ratio ( Sonda & Hehl, 2006; Yang *et al.*, 2004). The requirement of these three processes for invasion is a reasonable explanation for their coordinated regulation. The sustained expression till the end of the gametocyte stage may also suggest that key genes involved in the processes are essential to the survival of the parasite at the gametocyte – gamete stage or at least, are important in the events leading to this transition which may be potential candidates for transmission drug targeting.

Only the mature gametocytes can undergo further development in the mosquito mid-gut. Gametocytes escape the red blood cells taken up in the blood meal by the mosquito to form gametes - the male gametocyte differentiating into eight sperm-like gametes involving three rounds of DNA replication and nuclear division which happens in the first 10 minutes. The female differentiates into a single spherical gamete. This is triggered by environmental factors, which include the drop in temperature, the rise in pH and the presence of activating factors in the new vector host. Between 10 minutes and 1 hour, fertilization occurs ( Landau & Gautret, 1998). Meiosis does not immediately occur after nuclear division resulting in an ookinete with a higher amount of DNA. This ookinete develops into a spherical, more motile ookinete with an apical complex for traversing the mid-gut epithelium after 24 hours ( Landau & Gautret, 1998). In MOS, 2 clusters of biological interest were identified. Clusters 4 (153 genes) showed induction of genes only after 16 hours. GO terms associated with this cluster include "peptidyl-arginine N-methylation" (GO:0035246), "regulation of cell proliferation" (GO:0042127), "protein processing involved in protein targeting to mitochondrion" (GO:0006627), "protein farnesylation" (GO:0018343) and "U6 snRNA 3'-end processing" (GO:0034477). Peptidyl-arginine N-methylation has been shown to regulate cellular process including RNA processing, transcriptional regulation, signal transduction, DNA repair and also plays a role in targeting proteins to the plastid. There are interesting results from studies employing peptidyl-arginine N-methylation inhibitors that call for further investigation for a drug strategy in malaria ( Bedford & Richard, 2005; Dillon *et al.*, 2012). Protein farnesylation involves lipid post-translational modification that occurs in eukaryotic cells. In higher eukaryotes, farnesylation of some proteins (notably GTPase Ras) play a role in cell signal transduction, vesicle trafficking, and cell cycle progression. Increased levels of these proteins can potentially lead to cancer for which inhibitors (PFTIs) have been designed. Investigators are currently researching the use of PFTIs for against eukaryotic pathogens including *Plasmodium* species ( Eastman *et al.*, 2006; Tamanoi *et al.*, 2001; Wiesner *et al.*, 2004). 3'-end processing of snRNA and ncRNA are important in RNA biogenesis. Genes in cluster 9 (258 genes) were induced between the 0 – 16 hour with a rapid decline in trend after this. GO terms associated with these clusters include "peptidyl-diphthamide biosynthetic process from peptidyl-histidine" (GO:0017183) and "vesicle docking involved in exocytosis" (GO:0006904). Diphtamide is a unique post-translationally modified histidine residue of the elongation factor 2 conserved in eukarotic cells. Although the biological function of diphtamide is still not clearly understood, diphtamide has a well-studied pathological application. It functions as recognition site by some toxins ( Su *et al.*, 2013). How diphtamide makes cells susceptible to exploitation by toxins should be investigated. The triggering of calcium-mediated signaling pathways within the sporozoite is thought to be necessary to induce exocytosis of molecules required for sporozoite invasion ( Carey *et al.*, 2014). To date, no specific *Plasmodium* calcium dependent serine/threonine protein kinase (CDPK) inhibitors have been discovered even though seemingly abundant in the genome.

### Genes involved in different biological processes share common regulatory elements

**Figure 2.**
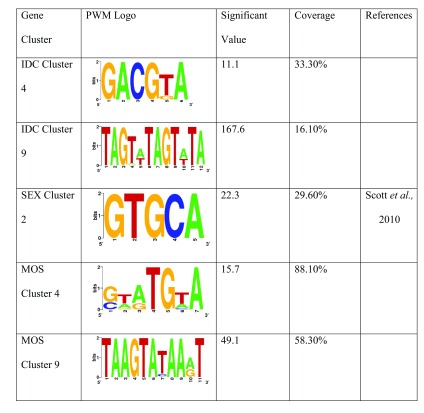
Logos of position weight matrices (PWM) identified in clusters of the IDC, SEX and MOS developmental stages of *Plasmodium berghei*. Significant values > 0 are relevant. References of logos identified in other research using other methods are highlighted in column 5.

Cis-regulatory elements are generally known to be located in regions upstream of the gene start codons. In this study, we searched for candidate motifs up to 800 bases upstream of each gene set for matching sequences using three different algorithms. From previous motifs that have been experimentally determined ( Painter *et al.*, 2011) a reliable candidate motif in the AT-rich and repetitive *Plasmodium* genome should have a reasonable degree of variability containing at least two or more bases other than A or T with a low occurrence in other locations in the *Plasmodium* genome ( Young *et al.*, 2008). Our study shows five candidate motifs for cis-regulatory elements obtained after analysis of clusters of each of the developmental stages of the parasite. The attributes of each motif is illustrated by the position weight matrices (PWM) logos, one of which is corroborated by an independent research carried out by a separate set of scientists employing a different method in Figure 2. As previously discussed, clustering of genes with similar expression patterns revealed 14 biological processes and pathways which possibly played integral roles in distinct stages of its developmental life-cycle of the *Plasmodium* species in response to transduced stimuli that lead to regulation of gene expression in the nucleus as a result of changes in its environments. The paucity of identified regulatory elements in *Plasmodium* is largely attributed to the nature of its AT-rich and highly repetitive genome. This will suggest that genes involved in these otherwise unrelated processes possibly share common regulatory sequences. This provides a possible explanation to how *Plasmodium* can coordinate complex responses to environmental changes with limited regulatory routes.

Data of cluster analysis of *Plasmodium* RNA-seq time-course data identifying stage-specific co-regulated biological processes and regulatory elements The raw data of cluster analysis Plasmodium RNA-seq time-course data are provided. The readme file contains descriptions for each data file. Click here for additional data file. Copyright: © 2016 Ashano E et al. 2016 Data associated with the article are available under the terms of the Creative Commons Zero "No rights reserved" data waiver (CC0 1.0 Public domain dedication).

## Conclusion

In this study analyzed *Plasmodium* RNA-seq data covering three life developmental stages to identify biological processes as possible candidates for drug targeting and their respective points of coordinated transcription control. To achieve this goal, we used an unsupervised machine learning approach, grouping genes which showed similar expression patterns across time-point for each respective developmental stage of the parasite. We showed that each development stage activated biological process custom to the anticipated environment unique to each development stage in which we identified 14 biological processes that may be integral at different stages of the parasites development, 11 of which have not been investigated in drug-related research. These include sustained upregulated biological processes linked to invasion *e.g.* peptidyl-diphathamine biosysthesis and protein processes involved in mitochondria targeting which could be targeted when designing drugs that prevent transmission. In agreement with other studies, the IDC expressed the fewest genes, a possible survival adaptation of the parasite, which makes targeting that stage difficult. In addition to these, some uncharacterized genes were also identified that may yet yield new drug targets following further investigations. We also showed in our study that genes involved in more than one biological process may share common regulatory elements which may explain the paucity of transcription factors unique to the species. We elucidated five such consensus sequences, four of which are novel sequences that may be potential cis-regulatory elements involved in coordinated control pending further validation studies.

## Data availability

The data referenced by this article are under copyright with the following copyright statement: Copyright: © 2016 Ashano E et al.

Data associated with the article are available under the terms of the Creative Commons Zero "No rights reserved" data waiver (CC0 1.0 Public domain dedication). 

Data files are openly available at *https://github.com/efejiroe/pb-tc-cluster-experiment-suppdata.*

F1000Research: Dataset 1. Data of cluster analysis of *Plasmodium* RNA-seq time-course data identifying stage-specific co-regulated biological processes and regulatory elements, 10.5256/f1000research.9093.d131414 ( Ashano *et al.*, 2016).
