# Supplementary material for: Cluster analysis of Plasmodium RNA-seq time-course data identifies stage-specific co-regulated biological processes and regulatory elements
Source: F1000Res. 2016 Aug 8;5:ISCB Comm J-1932. [Version 1] doi: 10.12688/f1000research.9093.1 (PMC5155496; doi:10.12688/f1000research.9093.1)
Supplement: Peer review history

# Peer review: Referee response for version 1

**DOI:** 10.5256/f1000research.9787.r17785

**Published:** 2016-12-09

**Authors:** Thomas D. Otto, Gavin Rutledge

**Affiliations:** 1 Wellcome Trust Sanger Institute, Hinxton, UK

Summary

 Ashano *et al *apply a clustering method for a reanalysis of a published dataset. The dataset is split into three groups and in each they identify genes that exhibit significant upregulation of gene expression and then cluster genes by RNA-seq gene expression profile. They identify clusters that exhibit stage specific upregulation and show that certain clusters have enriched GO terms associated with processes specific to the life cycle stage. Finally, they search for sequence motifs that are enriched in the upstream region of genes in a specific cluster, suggesting that these motifs may be regulatory elements for the clusters.

Review Comments

 The main criticism is the difficulty to understand the clustering, which is central to the paper. We believe that the clustering may contain fundamental flaws that are difficult to pinpoint due to the unclarity of the methods.

- It seems that the six different time points are split into three groups IDX, SEX and MOS. This is unintuitive and discards genes expressed at different stages.
- How robust is the clustering using maSigPro to different parameters?
- How was 9 clusters chosen?
- Does a different clustering algorithm yield similar results?
- The explanation of how genes are determined as being significantly upregulated and how the clustering is performed is unclear at the moment. Some more explanation would be useful. Particularly, it is not clear if only significantly upregulated genes are clustered or not, as later in the manuscript the authors refer to clusters with significantly upregulated genes suggesting that non-significant genes are also clustered.
- Better visualization of the clusters would be nice. For example, how many clusters are there and how many genes are in each of the clusters? In figure 1, it would be good to plot the expression profiles of all the genes in each of the clusters in order to get an idea of the variance in gene expression in each of the clusters.
- When describing the proportion of genes upregulated in each of the life-cycle stages, it would be useful to compare these results to other published datasets to look at the overlap.

Therefore the clustering results are unexpected. Figure 1 should show all the time points. We are not sure what the 0h time point refers to. Further just 1448 genes seem to be differentially expressed in the IDX, but this is in contrast to around 3300 genes in the IDX expressed in several *P. falciparum* studies ^1^^-^^3^.

 As the title of the manuscript suggests an analysis of *Plasmodium*, why choose a dataset that only has 3 idx timepoints, compared to several other RNA-Seq datasets with at least 7 timepoints, or over 48 timepoints in the above mentioned microarray experiments. There are a lot of published data, see plasmoDB. So it is unclear why this choice was made. In the absence of a rational reason, it would be good to use an alternative dataset to get better resolution.

 Last, we do not understand how 4383 genes out of 5003 can be differentially expressed in the MOS stage, Table 1 of the manuscript. In the figure 2 of the paper presenting the used date ^4^, the amount of genes expressed in the ookinete stage is similar to that of the other stages. Could you please explain the differences.

 Better data visualization and more explicit numbers quoted in a more thorough walkthrough of the methods would help clear up these questions.

 If the authors choose not to include further datasets, the title or at least the abstract should specify that the analysis was done on *P. berghei*. The manuscript currently generalizes the findings in this species to be generally valid for all other *Plasmodium* species.

 The introduction is very general, and could give a better overview of similar work performed in malaria recently.

 In the GO enrichment, genes that are not expressed in the dataset should be excluded from the gene background (which is not possible in plasmoDB). It would be better to use topGO. Also, the GO terms are from geneDB and not from the annotation center.

 Why mention SEX cluster 5 in the GO term section if there are no significant GO terms?

 Upstream regions of genes for the motif identification were obtained from *P. falciparun* orthologs. Why not use *P. berghei* upstream regions, seeing as the RNA-seq data is from *P. berghei*?

 In the results it is stated that motifs were searched in the 800bp upstream region of each gene, but in the methods it says 1000bp. Which is correct?

 Additional information on the identified motifs would be nice. For example, distribution of the motifs in the upstream region, presence of the motifs in upstream regions of other genes, etc. Additional comparison of the motifs to motifs identified in other studies would also be good, for example known AP2 binding motifs, with a lot of work having been performed by Ilinas ^5^.

   

 It is highly speculative to state that a lower number of genes are upregulated in the IDC stage due to immune pressure. The mosquito also possesses an immune system that would likely exhibit similar pressure.

   

 In table 2, the background percent column would benefit from better explanation and explicit numbers: ie. 5 genes out of 10 instead of just 50%. P-values might also be useful to include here. 

 In many cases references are placed at the end of paragraphs instead of after the particular statement that the references support, for example in the lipoate paragraph.

 Last, we would encourage the authors to discuss their finding with other similar papers in the field, especially the found motifs and the upregulated gene clusters.

 There are multiple spelling mistakes and strangely formulated sentences throughout the manuscript that hinder understanding.

We have read this submission. We believe that we have an appropriate level of expertise to confirm that it is of an acceptable scientific standard, however we have significant reservations, as outlined above.

## References

[1] BozdechZLlinásMPulliamBLWongEDZhuJDeRisiJL: The transcriptome of the intraerythrocytic developmental cycle of Plasmodium falciparum. *PLoS Biol*.2003;1(1) : 10.1371/journal.pbio.0000005 E5 10.1371/journal.pbio.0000005 12929205PMC176545

[2] LlinásMBozdechZWongEDAdaiATDeRisiJL: Comparative whole genome transcriptome analysis of three Plasmodium falciparum strains. *Nucleic Acids Res*.2006;34(4) : 10.1093/nar/gkj517 1166-73 10.1093/nar/gkj517 16493140PMC1380255

[3] OttoTDWilinskiDAssefaSKeaneTMSarryLRBöhmeULemieuxJBarrellBPainABerrimanMNewboldCLlinásM: New insights into the blood-stage transcriptome of Plasmodium falciparum using RNA-Seq. *Mol Microbiol*.2010;76(1) : 10.1111/j.1365-2958.2009.07026.x 12-24 10.1111/j.1365-2958.2009.07026.x 20141604PMC2859250

[4] OttoTDBöhmeUJacksonAPHuntMFranke-FayardBHoeijmakersWAReligaAARobertsonLSandersMOgunSACunninghamDErhartABillkerOKhanSMStunnenbergHGLanghorneJHolderAAWatersAPNewboldCIPainABerrimanMJanseCJ: A comprehensive evaluation of rodent malaria parasite genomes and gene expression. *BMC Biol*.2014;12: 10.1186/s12915-014-0086-0 86 10.1186/s12915-014-0086-0 25359557PMC4242472

[5] CampbellTLDe SilvaEKOlszewskiKLElementoOLlinásM: Identification and genome-wide prediction of DNA binding specificities for the ApiAP2 family of regulators from the malaria parasite. *PLoS Pathog*.2010;6(10) : 10.1371/journal.ppat.1001165 e1001165 10.1371/journal.ppat.1001165 21060817PMC2965767

# Peer review: Referee response for version 1

**DOI:** 10.5256/f1000research.9787.r17942

**Published:** 2016-11-24

**Authors:** Hilary Ann Coller

**Affiliations:** 1 Department of Molecular, Cell and Developmental Biology (MCDB), University of California, Los Angeles (UCLA), Los Angeles, CA, USA

This manuscripts describes a carefully executed analysis of gene expression patterns over the Plasmodium life cycle. The authors have identified genes and GO categories that are upregulated at different stages of the Plasmodium life cycle, the intraerythrocytic developmental cycle, the gamete-fertilization stage and the sexual development stage. The genes that are upregulated suggest some pathways that may be targeted as new drugs to combat malaria. The authors also identified five motifs that are enriched in the promoters of the genes regulated at the different stages. Identifying enriched motifs is especially challenging in these species because of their AT-rich genomes. The title and abstract are accurate reflections of the work. The experimental design is careful and well-described. The conclusions are appropriate. The work relies on data that are already in the public domain.

I have read this submission. I believe that I have an appropriate level of expertise to confirm that it is of an acceptable scientific standard.

# Peer review: Referee response for version 1

**DOI:** 10.5256/f1000research.9787.r15551

**Published:** 2016-09-14

**Authors:** Manuel Corpas

**Affiliations:** 1 The Genome Analysis Centre, Norwich, UK

The article "Cluster analysis of Plasmodium RNA-seq data identifies stage-specific co-regulated biological processes and regulatory elements" uses an RNA-seq dataset published in Otto et al. ^1^ for a number of Plasmodium species. Authors use the assumption that co-transcribed genes are likely to be controlled by a common regulatory element. Subsequently they provide a clustering of expression profiles using that dataset.

 I would propose the following enhancements to the article:

1. In order to ensure the reproducibility of the experiment, I would request if more information could be provided about the raw dataset from which the study has derived. Things like number of samples, what conditions, how big the data is, etc. This information should be available in the source paper.
2. What parameters did you use to run maSigPro? What parameters did you use to cluster gene expression patterns?
3. I am concerned about the use of Herrero et al.'s tool, which was published in 2001. This tool was developed for array data, a long time before NGS data like transcriptomics became available.
4. In results: 155 genes out of 1448 is not ~0.1%. It's ~10%.
5. In table 2, I am not sure what the background percentages are. I would appreciate if legends made figures self explanatory.
6. When presenting results, for each section there is a lot of background information. For example:
  Section 1: "Characterized genes may provide alternative sources for drug targets of the IDC". I find this header too obvious and uninformative. The text at the beginning of this section up to the sentence "For the ICD, to clusters [...]" is all material that would be better placed in a discussion section. Clearly this text is not part of what I would call "results".
  I find the other sections within Results also have a lot of preliminary background information that in my opinion would be more suitable for a discussion section than results. Another example is the section "Sustains up-regulated [...]". I still think this background information is necessary though to understand the context of results.
7. I would find it helpful if Figure 3 was referenced from the text in the relevant section. Currently, as it appears in the paper, I had no idea why of how it related to the paper until I read the section "Sustained up-regulated biological processes linked to invasion may present key drug targets to prevent transmission".
8. In conclusions I don’t understand this sentence:
  “We showed that each development stage activated biological process custom to the anticipated environment unique to each development stage in which we identified 14 biological processes that may be integral at different stages of the parasites development, 11 of which have not been investigated in drug-related research."

I have read this submission. I believe that I have an appropriate level of expertise to confirm that it is of an acceptable scientific standard, however I have significant reservations, as outlined above.

## References

[1] OttoTDBöhmeUJacksonAPHuntMFranke-FayardBHoeijmakersWAReligaAARobertsonLSandersMOgunSACunninghamDErhartABillkerOKhanSMStunnenbergHGLanghorneJHolderAAWatersAPNewboldCIPainABerrimanMJanseCJ: A comprehensive evaluation of rodent malaria parasite genomes and gene expression. *BMC Biol*.2014;12: 10.1186/s12915-014-0086-0 86 10.1186/s12915-014-0086-0 25359557PMC4242472
